# A retrospective comparative exploratory study on two Methylentetrahydrofolate Reductase (MTHFR) polymorphisms in esophagogastric cancer: the A1298C MTHFR polymorphism is an independent prognostic factor only in neoadjuvantly treated gastric cancer patients

**DOI:** 10.1186/1471-2407-14-58

**Published:** 2014-02-03

**Authors:** Susanne Blank, Sivaramakrishna Rachakonda, Gisela Keller, Wilko Weichert, Florian Lordick, Rupert Langer, Christoph Springfeld, Thomas Bruckner, Karen Becker, Rajiv Kumar, Katja Ott

**Affiliations:** 1 Department of Surgery, University Hospital of Heidelberg, Im Neuenheimer Feld 110, Heidelberg 69120, Germany; 2 DKFZ, University of Heidelberg, Heidelberg, Germany; 3 Institute of pathology, Technische Universitaet, Muenchen, Munich, Germany; 4 Institute of pathology, University of Heidelberg, Heidelberg, Germany; 5 University Cancer Center Leipzig (UCCL), Leipzig, Germany; 6 Institute of pathology, University of Bern, Bern, Switzerland; 7 National Center of Tumor Diseases, University of Heidelberg, Heidelberg, Germany; 8 Medical Biometry, University of Heidelberg, Heidelberg, Germany

**Keywords:** Esophagogastric adenocarcinoma, Prognostic factors, Folate metabolism, Methylentetrahydrofolate reductase, Genetic polymorphisms, C677T, A1298C

## Abstract

**Background:**

Methylentetrahydrofolate reductase (MTHFR) plays a major role in folate metabolism and consequently could be an important factor for the efficacy of a treatment with 5-fluorouracil. Our aim was to evaluate the prognostic and predictive value of two well characterized constitutional MTHFR gene polymorphisms for primarily resected and neoadjuvantly treated esophagogastric adenocarcinomas.

**Methods:**

569 patients from two centers were analyzed (gastric cancer: 218, carcinoma of the esophagogastric junction (AEG II, III): 208 and esophagus (AEG I): 143). 369 patients received neoadjuvant chemotherapy followed by surgery, 200 patients were resected without preoperative treatment. The MTHFR C677T and A1298C polymorphisms were determined in DNA from peripheral blood lymphozytes. Associations with prognosis, response and clinicopathological factors were analyzed retrospectively within a prospective database (chi-square, log-rank, cox regression).

**Results:**

Only the MTHFR A1298C polymorphisms had prognostic relevance in neoadjuvantly treated patients but it was not a predictor for response to neoadjuvant chemotherapy. The AC genotype of the MTHFR A1298C polymorphisms was significantly associated with worse outcome (p = 0.02, HR 1.47 (1.06-2.04). If neoadjuvantly treated patients were analyzed based on their tumor localization, the AC genotype of the MTHFR A1298C polymorphisms was a significant negative prognostic factor in patients with gastric cancer according to UICC 6^th^ edition (gastric cancer including AEG type II, III: HR 2.0, 95% CI 1.3-2.0, p = 0.001) and 7^th^ edition (gastric cancer without AEG II, III: HR 2.8, 95% CI 1.5-5.7, p = 0.003), not for AEG I. For both definitions of gastric cancer the AC genotype was confirmed as an independent negative prognostic factor in cox regression analysis. In primarily resected patients neither the MTHFR A1298C nor the MTHFR C677T polymorphisms had prognostic impact.

**Conclusions:**

The MTHFR A1298C polymorphisms was an independent prognostic factor in patients with neoadjuvantly treated gastric adenocarcinomas (according to both UICC 6^th^ or 7^th^ definitions for gastric cancer) but not in AEG I nor in primarily resected patients, which confirms the impact of this enzyme on chemotherapy associated outcome.

## Background

Multimodal treatment is the standard of care for locally advanced adenocarcinomas of the esophagus or stomach since several randomized trials and meta-analyses have shown a prognostic benefit for (peri-) preoperative therapy versus surgery alone [1-4]. For patients who received multimodal treatment it is widely accepted that responding patients have a significantly better outcome than nonresponding patients [5-8]. However, depending on the therapy regimen applied, only 25-50% of patients respond to (peri-) preoperative treatment. Until now no molecular markers are available to predict response or survival in clinical routine and to tailor treatment individually. Despite the current guidelines favoring a multimodal treatment for locally advanced tumors, a relevant number of patients are still resected without preoperative treatment due to favorable tumor categories, differing local standards, individual risk factors or patients’ choice.

Simple pretherapeutically available cliniocopathological factors like tumor localization, grading, content of signet ring cells and Laurén classification have been found to be associated with prognosis in patients with and without preoperative treatment in several studies [9-14]. So far, however these factors are not routinely used to tailor treatment or to stratify groups within clinical trials. Several studies on molecular and genetic prognostic and/or predictive markers in patients with gastric cancer have been published, but none of them (apart from HER-2 [15] in the palliative setting) gained clinical relevance [16,17]. Aside from tumor related factors, constitutional factors such as genetic polymorphisms are accepted to be associated with response and prognosis in adenocarcinomas of esophagus or stomach [18-21]. Most data exist for genetic polymorphisms being involved in pharmacodynamics and drug metabolism. Our own data on constitutional polymorphisms have shown to a large extent that the examined constitutional variants were associated rather with prognosis, than with response to preoperative treatment [22,23]. Only a few studies have investigated constitutional factors with the goal to clarify whether these factors are only relevant in the presence of chemotherapy or if they are prognostic factors irrespective of preoperative treatment [22].

Methylentetrahydrofolate reductase (MTHFR) plays a major role in the folate metabolism and consequently could be an important factor for the efficacy of a treatment with 5-fluorouracil (5-FU) [24-27].

The MTHFR single nucleotide polymorphism (SNP) C677T (rs 1801133) has shown to be associated with prognosis in gastric cancer patients in several studies [23,28,29], however results are still conflicting [30] and the clinical relevance of this SNP has to be reproduced before clinical consequences can be drawn.

Another SNP in the MTHFR gene (A1298C) (rs 1801131) has also been investigated in several studies but results are less convincing than for C677T [31,32]. A recent study of our group found a significant association of this MTHFR gene A1298C polymorphism with prognosis in 258 neoadjuvantly treated esophagogastric adenocarcinomas [23].

In this study we focused on these two polymorphisms in the MTHFR gene to confirm the prognostic significance of the respective MTHFR gene polymorphisms in a larger group of esophagogastric cancer patients with neoadjuvant treatment as well as to test whether these polymorphisms can be used as a predictor for response to neoadjuvant chemotherapy. Additionally we wanted to test their relevance in a group of patients without perioperative chemotherapy.

## Methods

**Figure 1:**
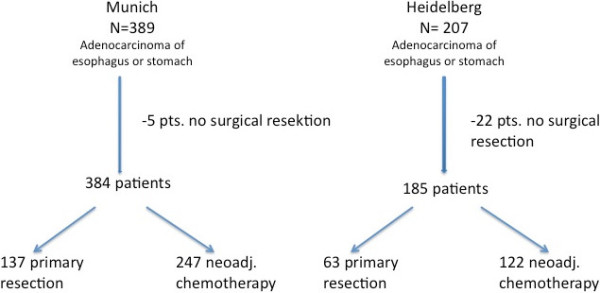
Flow chart.

569 patients with histologically proven adenocarcinoma of the esophagus (AEG I), esophagogastric junction (AEG II, III) or stomach (GC) were included in the study. Patients were treated in the Klinikum rechts der Isar, Munich from 1994–2005 (n = 361) or the University Hospital Heidelberg, Surgical Department from 2007–2010 (n = 235), 27 patients were excluded due to non-operative treatment. 369 patients received neoadjuvant chemotherapy followed by surgery, while 200 patients were resected without preoperative treatment (Figure 1).

It is of note that for 244 of the 369 neoadjuvantly treated patients the results with shorter follow-up have been reported recently for both analyzed polymorphisms [23].

The study was approved by the ethical committee of the University of Heidelberg and of the Technical University of Munich and written informed consent was obtained from all patients.

### Data assessment

Demographic data, primary tumor localization, grading, type of resection, Lauren’s subtype, ypTNM- and R-category, data on clinical and histopathological response as well as perioperative complications and mortality were documented prospectively in a database containing all patients with carcinoma of the esophagus or stomach in the two centers.

### Genotyping

Blood was collected from patients before surgery. DNA from blood was isolated with Qiagen mini-preparation kits and genotyped for the two polymorphisms in the MTHFR gene (C677T and A1298C). Genotyping was performed using KASPar chemistry, a competitive allele-specific PCR genotyping system (http://www.lgcgenomics.com) according to manufacturer’s instructions. The PCR was carried out in plates of 96 wells, in a total reaction volume of 8 μl using 10 ng of genomic DNA, 2 μl 2X KASPar reaction mix and 0.11 μl of the assay mix. The PCR conditions were: an initial denaturation at 94°C for 15 minutes, 10 touch-down cycles of 20 s at 94°C and 60 s at 61-55°C (temperature decrement by 0.6°C per cycle) and additional 25–30 cycles at 20 s at 94°C and 60 s at 55°C. Genotypes in amplified products were determined by differences in VIC and FAM fluorescent level in plate read operation on ABI PRISM 7900HT (Applied Biosystems, Foster City, CA) using SDS 2.2 Software. Post operation data were transferred to Microsoft Excel files and converted into genotype information. The genotype quality control was validated through DNA sequencing in 5% of the samples.

### Chemotherapy

369 patients were treated with neoadjuvant chemotherapy: Munich (n = 247): OLF/PLF regimen: either oxaliplatin 85 mg/m^2^ or cisplatin 50 mg/m^2^ on days 1,15,29 and folinic acid (500 mg/m^2^ over 2 h) plus fluorouracil (2000 mg/m^2^) on days 1,8,15,22,29 and 36, all repeated on day 49, for patients with a good health status additionally paclitaxel (80 mg/m^2^) on days 0, 14 and 28) was given [33]. Heidelberg (n = 122): EOX-regimen: epirubicin 50 mg/m2 (day 1), oxaliplatin 130 mg/m2 (day 1), and capecitabin 1,250 mg/m2 (days 1–21), all repeated on day 22., PLF (see above), and FLOT: oxaliplatin 85 mg/m2 (day 1), docetaxel 50 mg/m2 (day 1), folinacid 200 mg/m2 (day 1), and 5-fluoruracil 2,600 mg/m2 (day 1), all repeated on day 15 [9].

### Surgery

The type of surgery was performed according to the tumor localization and local standards: for patients with AEG I either an abominothoracic approach with intrathoracic anastomosis (Ivor Lewis procedure) [34] or a transhiatal esophagectomy [35], both including an abdominal D-2 lymphadenectomy, for patients with adenocarcinoma of the esophagogastric junction (AEG II, III) a transhiatal gastrectomy, in some cases an Ivor lewis procedure [36] or a transhiatal esophagectomy [35] was performed. Patients with gastric cancer received a total gastrectomy or subtotal gastrectomy [37] if an adequate proximal resection margin was possible. Both procedures included a D2-lymphadenectomy.

### Response to chemotherapy

Clinical response was assessed after chemotherapy and before surgery comparing pre- and posttherapeutic computed tomography imaging and endoscopy. The evaluation was done by an interdisciplinary tumor board of the Klinikum rechts der Isar, Munich or by the Surgical Department of the University of Heidelberg. Criteria for response were a decrease of the maximal transversal tumor diameter of >50% in CT and an estimated decrease in endoluminal tumor size of >75% in endoscopy. Patients with minor response, no change or progressive disease were classified as nonresponder [38-40].

Histopathological response was assessed according to the Becker regression score [5,41]: tumor regression grade (TRG) 1a (complete regression) and 1b (<10% residual tumor) were classified as histopathological response, TRG 2 (10-50% residual tumor) and 3 (>50% residual tumor) as nonresponse.

### Follow-up

Follow-up was done according to the local guidelines. Patients who were not followed in one of the two centers were contacted by phone to obtain follow-up data. Median time of follow-up of the surviving patients was 41.1+/− 25.8 months.

### Statistical analysis

For statistical analysis we used SPSS 20.0 (IBM Inc. Chicago). Quantitative data is presented as mean +/− standard deviation. Survival curves were estimated by the Kaplan-Meier method and presented in months from time of diagnosis to death. Differences in survival times were calculated using the log-rank test. For univariate and multivariate analysis we used the Cox proportional hazard model. For correlation between different parameters we used the Chi-square-test (two-sided) where appropriate. P-values <0.05 were considered as statistically significant.

## Results

230 patients out of 569 have died. Median survival of the entire population was 66.0 months. Median survival of patients treated in Munich was 72.8 months, in Heidelberg 85.6 months.

Patients’ characteristics including survival times are presented in Table 1.

**Table 1:** Patients’ characteristics and survival times according to clinicopathological factors

**Characteristics**		**n**	**%**	**Median (months)**	**3-Y-S (%)**	**5-Y-S (%)**	**p**
a: All patients							
**Sex**	Male	431	75.7	66.0	60.8	52.6	0.637
	Female	138	24.3	66.0	57.0	52.7	
**Localization**	AEG	351	61.7	74.2	60.4	52.4	0.81
**UICC 7th edition**	Gastric cancer	212	37.3	62.2	58.7	52.3	
**Localization**	AEG I	143	25.1	108.0	63.0	55.5	0.288
**UICC 6th edition**	AEG II/III + GC	426	74.9	64.1	58.9	51.8	
**Laurén**	intestinal	320	56.2	78.8	65.1	57.2	0.029
	non-intestinal	234	41.1	47.1	54.8	48.2	
**Grading**	low grade	166	29.2	108.0	69.3	64.7	0.012
	high grade	395	69.4	54.1	56.4	47.8	
**pT**	pT0	38	6.7	n.r.	85.6	76.4	<0.001
	pT1	52	9.1	n.r.	83.5	83.5	
	pT2	276	48.5	85.6	68.7	62.5	
	pT3	184	32.2	26.7	37.4	25.4	
	pT4	19	3.3	14.2	5.3	0.0	
**pN**	pN0	242	42.5	101.9	76.8	69.6	<0.001
	pN1	201	35.3	33.3	48.2	39.4	
	pN2	74	13	26.3	41.3	34.6	
	pN3	28	4.9	18.9	25.4	25.4	
	pNx	24	4.2	n.r.	78.5	78.5	
**pM**	pM0	525	92.3	72.8	62.0	54.6	0.001
	pM1	44	7.7	23.6	36.3	32.2	
**R**	R0	476	83.7	74.2	64.7	56.7	<0.001
	R1/R2	93	16.3	20.3	34.2	32.2	
**Neoadjuvant chemotherapy**	yes	369	64.9	74.2	61.5	52.6	0.157
	no	200	35.1	62.2	56.8	53.1	
**Characteristics**		**n**	**%**	**Median (months)**	**3-Y-S (%)**	**5-Y-S (%)**	**p**
b: Neoadjuvantly treated patients							
**Sex**	Male	309	83.7	78.8	63.2	53.7	0.253
	Female	60	16.3	48.6	53.0	46.9	
**Localization**	AEG	287	77.8	78.8	63.4	54.4	0.259
**UICC 7th edition**	Gastric cancer	82	22.2	42.5	55.7	46.3	
**Localization**	AEG I	139	37.7	108.0	62.8	55.3	0.653
**UICC 6th edition**	AEG II/III + GC	230	62.3	66.0	61.0	51.1	
**Laurén**	Intestinal	220	59.6	108.0	67.3	58.7	0.014
	Non-intestinal	140	37.9	40.5	54.4	44.6	
**Grading**	Low grade	109	29.5	108.0	73.0	68.2	0.008
	High grade	254	68.8	47.1	56.8	45.3	
**pT**	pT0	38	10.3	n.r.	85.6	76.4	<0.001
	pT1	35	9.5	n.r.	83.2	83.2	
	pT2	169	45.8	101.9	68.7	60.3	
	pT3	116	31.4	26.9	39.5	25.2	
	pT4	11	3.0	14.2	0.0	0.0	
**pN**	pN0	157	42.5	108.0	77.4	69.3	<0.001
	pN1	142	38.5	34.0	49.0	38.6	
	pN2	39	10.6	29.1	44.1	31.6	
	pN3	17	4.6	18.9	38.5	38.5	
	pNx	14	3.8	n.r.	77.9	77.9	
**pM**	pM0	328	88.9	82.7	65.1	55.6	<0.001
	pM1	41	11.1	23.6	33.4	28.6	
**R**	R0	291	78.9	n.r.	69.0	58.4	<0.001
	R1/R2	78	21.1	20.3	32.7	30.4	
**Clinical response**	Responder	105	28.5	39.2	82.6	74.2	<0.001
	Nonresponder	263	71.3	108.0	52.4	42.8	
**TRG**	1a,1b	104	28.2	n.r.	83.8	77.4	<0.001
	2,3	263	71.3	39.2	52.5	42.1	
**Characteristics**		**n**	**%**	**Median (months)**	**3-Y-S (%)**	**5-Y-S (%)**	**p**
c: Primarily resected patients							
**Sex**	Male	122	61.0	62.2	54.6	50.1	0.318
	Female	78	39.0	66.0	60.5	57.5	
**Localization**	AEG	64	32.0	32.6	46.9	44.3	0.049
**UICC 7th edition**	Gastric cancer	131	65.5	64.1	60.7	56.4	
**Localization**	AEG I	4	2.0	n.r.	75.0	75.0	0.619
**UICC 6th edition**	AEG II/III + GC	196	98.0	62.2	56.5	52.9	
**Laurén**	Intestinal	100	50.0	62.2	59.8	53.4	0.807
	Non-intestinal	94	47.0	64.1	55.7	54.0	
**Grading**	Low grade	57	28.5	62.2	61.5	55.9	0.526
	High grade	141	70.5	64.1	55.8	52.2	
**pT**	pT0	0	0.0				<0.001
	pT1	17	8.5	n.r.	86.5	86.5	
	pT2	107	53.5	85.6	68.5	65.4	
	pT3	68	34.0	25.8	33.2	26.6	
	pT4	8	4.0	10.3	12.5	12.5	
**pN**	pN0	85	42.5	85.6	76.0	70.2	<0.001
	pN1	59	29.5	32.0	45.4	41.6	
	pN2	35	17.5	21.7	37.9	28.4	
	pN3	11	5.5	16.1	9.1	9.1	
	pNx	10	5.0	n.r.	78.8	78.8	
**pM**	pM0	197	98.5	62.2	56.6	52.9	0.684
	pM1	3	1.5	n.r.	66.7	66.7	
**R**	R0	185	92.5	64.1	57.8	53.9	0.144
	R1/R2	15	7.5	18.1	44.4	0.0	

MS = median survival, 3-Y-S = 3-Year-Survival, 5-Y-S = 5-Year-Survival, AEG = adenocarcinoma of the esophagogastric junction, TRG = tumor regression grade, n.r. = not reached.

Relevant prognostic factors were Laurén’s type (p = 0.029), Grading (p = 0.012), (y)pT-category (p < 0.0000001), (y)pN-category (p < 0.0000001), (y)pM-category (p = 0.001), R-category (p < 0.0000001), additionally clinical response (p < 0.0000001) and histopathological response (p < 0.0000001) for patients treated with neoadjuvant chemotherapy.

### Genotype frequencies and correlation with clinicopathological factors

The genotype frequencies were in accordance with the Hardy-Weinberg-equilibrium. Frequencies of the individual genotypes are presented in Tables 2 and 3. No individual genotype showed any statistical correlation with a clinicopathological factor listed above, also if analyzed separately for the respective tumor entities (AEG versus GC) and treatment groups (primary resection versus neoadjuvant treatment).

**Table 2:** Genotype frequencies: MTHFR C677T

		** MTHFR C677T**			**p**
		** CC**	** CT**	** TT**	
**All patients**		254 (44.6%)	262 (46.0%)	53 (9.3%)	
**Status**	Alive	143 (42.2%)	162 (47.8%)	34 (10%)	0.342
	Dead	111 (48.3%)	100 (43.5%)	19 (8.3%)	
**Sex**	Male	189 (43.9%)	202 (46.9%)	40 (9.3%)	0.775
	Female	65 (47.1%)	60 (43.5%)	13 (9.4%)	
**Localization**	AEG	153 (43.6%)	169 (48.1%)	29 (8.3%)	0.337
**UICC 7th edtiion**	Gastric cancer	99 (46.7%)	90 (42.5%)	23 (10.8%)	
**Localization**	AEG I	63 (44.1%)	68 (47.6%)	12 (8.4%)	0.868
**UICC 6th edition**	AEG II/III + GC	191 (44.8%)	194 (45.5%)	41 (9.6%)	
**Laurén**	Intestinal	137 (42.8%)	151 (47.2%)	32 (10.0%)	0.731
	Non-intestinal	107 (45.7%)	107 (45.7%)	20 (8.5%)	
**Grading**	Low grade	74 (44.6%)	75 (45.2%)	17 (10.2%)	0.89
	High grade	174 (44.1%)	185 (46.8%)	36 (9.1%)	
**pT**	pT0	14 (36.8%)	21 (55.3%)	3 (7.9%)	0.751
	pT1	20 (38.5%)	28 (53.8%)	4 (7.7%)	
	pT2	134 (48.6%)	118 (42.8%)	24 (8.7%)	
	pT3	78 (42.4%)	86 (46.7%)	20 (10.9%)	
	pT4	8 (42.1%)	9 (47.4%)	2 (10.5%)	
**pN**	pN0	109 (45.0%)	115 (47.5%)	18 (7.4%)	0.642
	pN1	93 (46.3%)	88 (43.8%)	20 (10.0%)	
	pN2	31 (41.9%)	33 (44.6%)	10 (13.5%)	
	pN3	12 (42.9%)	15 (53.6%)	1 (3.6%)	
	pNx	9 (37.5%)	11 (45.8%)	4 (16.7%)	
**pM**	pM0	236 (45.0%)	243 (46.3%)	46 (8.8%)	0.292
	pM1	18 (40.9%)	19 (43.2%)	7 (15.9%)	
**R**	R0	213 (44.7%)	218 (45.8%)	45 (9.5%)	0.948
	R1/R2	41 (44.1%)	44 (47.3%)	8 (8.6%)	
**Neoadjuvant chemotherapy**	yes	161 (43.6%)	179 (48.5%)	29 (7.9%)	0.135
	no	93 (46.5%)	83 (41.5%)	24 (12.0%)	
**Clinical response**	Responder	44 (41.9%)	52 (49.5%)	9 (8.6%)	0.885
	Nonresponder	117 (44.5%)	126 (47.9%)	20 (7.6%)	
**TRG**	1a,1b	44 (42.3%)	53 (51.0%)	7 (6.7%)	0.783
	2,3	116 (44.1%)	125 (47.5%)	22 (8.4%)	

AEG = adenocarcinoma of the esophagogastric junction, GC = gastric cancer, TRG = tumor regression grade.

**Table 3:** Genotype frequencies: MTHFR A1298C

		** MTHFR A1298C**			**p**
		** AA**	** AC**	** CC**	
**All patients**		244 (42.9%)	268 (47.1%)	54 (9.5%)	
**Status**	Alive	152 (45.1%)	149 (44.2%)	36 (10.7%)	0.162
	Dead	92 (40.2%)	119 (52.0%)	18 (7.9%)	
**Sex**	Male	184 (43.0%)	204 (47.1%)	40 (9.3%)	0.945
	Female	60 (43.5%)	64 (46.4%)	14 (10.1%)	
**Localization**	AEG	152 (43.7%)	164 (47.1%)	32 (9.2%)	0.793
**UICC 7th edtition**	Gastric cancer	87 (41.0%)	103 (48.6%)	22 (10.4%)	
**Localization**	AEG I	60 (42.6%)	68 (48.2%)	13 (9.2%)	0.968
**UICC 6th edition**	AEG II/III + GC	184 (43.3%)	200 (47.1%)	41 (9.6%)	
**Laurén**	Intestinal	140 (44.0%)	150 (47.2%)	28 (8.8%)	0.835
	Non-intestinal	100 (42.9%)	109 (46.8%)	24 (10.3%)	
**Grading**	Low grade	70 (42.4%)	77 (46.7%)	18 (10.9%)	0.76
	High grade	172 (43.8%)	186 (47.3%)	35 (8.9%)	
**pT**	pT0	16 (42.1%)	18 (47.4%)	4 (10.5%)	0.701
	pT1	17 (33.3%)	28 (54.9%)	6 (11.8%)	
	pT2	119 (43.3%)	125 (45.5%)	31 (11.3%)	
	pT3	83 (45.4%)	88 (48.1%)	12 (6.6%)	
	pT4	9 (47.4%)	9 (47.4%)	1 (5.3%)	
**pN**	pN0	98 (40.7%)	118 (49.0%)	25 (10.4%)	0.654
	pN1	97 (48.5%)	84 (42.0%)	19 (9.5%)	
	pN2	30 (41.1%)	37 (50.7%)	6 (8.2%)	
	pN3	9 (32.1%)	16 (57.1%)	3 (10.7%)	
	pNx	10 (41.7%)	13 (54.2%)	1 (4.2%)	
**pM**	pM0	226 (43.3%)	244 (46.7%)	52 (10.0%)	0.402
	pM1	18 (40.9%)	24 (54.5%)	2 (4.5%)	
**R**	R0	199 (42.0%)	226 (47.7%)	49 (10.3%)	0.239
	R1/R2	45 (48.9%)	42 (45.7%)	5 (5.4%)	
**Neoadjuvant chemotherapy**	Yes	156 (42.6%)	179 (48.9%)	31 (8.5%)	0.4
	No	88 (44.0%)	89 (44.5%)	23 (11.5%)	
**Clinical response**	Responder	46 (43.8%)	49 (46.7%)	10 (9.5%)	0.812
	Nonresponder	109 (41.9%)	130 (50.0%)	21 (8.1%)	
**TRG**	1a,1b	41 (39.4%)	55 (52.9%)	8 (7.7%)	0.628
	2,3	114 (43.8%)	123 (47.3%)	23 (8.8%)	

AEG = adenocarcinoma of the esophagogastric junction, GC = gastric cancer, TRG = tumor regression grade.

### Genotypes and survival

#### Survival and type of treatment

Survival analysis was conducted for the whole study cohort as well as for neoadjuvantly and primarily resected patients separately. The MTHFR C677T polymorphisms did not show a statistically significant influence on survival times in all patients (p = 0.412) nor in any analyzed subgroup (in neoadjuvantly treated patients p = 0.745, in primarily resected patients p = 0.295), although there was a slight trend for shorter survival of patients with the CC genotype (54.1 months median in patients with CC genotype versus 82.7 months in patients with CT and TT genotype, p = 0.237), Table 4a.

**Table 4:** Survival according to the individual genotypes

		**n**	**MS (months)**	**p**	**HR (95% CI)**
a: All patients					
**MTHFR C677T**	CC	254	54.1	0.412	
	CT	262	82.7		0.88 (0.67-1.15)
	TT	53	n.r.		0.75 (0.46-1.22)
	CC	254	54.1	0.237	
	CT + TT	315	82.7		0.86 (0.66-1.11)
**MTHFR A1298C**	AA	244	82.7	0.036	
	AC	268	47.1		1.31 (1.0-1.72)
	CC	54	74.2		0.77 (0.47-1.28)
	AC	268	47.1	0.016	
	AA + CC	301	82.7		0.73 (0.56-0.94)
		**n**	**MS (months)**	**p**	**HR (95% CI)**
b: Neoadjuvantly treated patients					
**MTHFR C677T**	CC	161	55.4	0.745	
	CT	179	82.7		0.98 (0.7-1.38)
	TT	29	n.r.		0.77 (0.4-1.51)
	CC	161	55.4	0.77	
	CT + TT	208	82.7		0.95 (0.69-1.32)
**MTHFR A1298C**	AA	156	101.9	0.063	
	AC	179	47.1		1.43 (1.02-2.01)
	CC	31	74.2		0.85 (0.44-1.67)
	AC	179	47.1	0.02	
	AA + CC	190	101.9		0.68 (0.49-0.94)
		**n**	**MS (months)**	**p**	**HR (95% CI)**
c: Primarily resected patients					
**MTHFR C677T**	CC	93	54.1	0.295	
	CT	83	n.r.		0.72 (0.45-1.15)
	TT	24	66.0		0.69 (0.34-1.4)
	CC	93	54.1	0.119	
	CT + TT	107	66.0		
**MTHFR A1298C**	AA	88	66.0	0.366	
	AC	89	47.6		0.16 (0.74-1.82)
	CC	23	62.2		0.68 (0.31-1.46)
	AC	89	47.6	0.295	
	AA + CC	111	66.0		0.79 (0.52-1.22)

In contrast, the MTHFR A1298C polymorphisms were prognostically relevant (p = 0.036) in all patients. The AC genotype was associated with a worse outcome compared to AA and CC. As we did not find a prognostic difference between the AA and CC genotype, the two homozygous genotypes were combined and analyzed together: 47.1 months median for AC genotype versus 82.7 months for AA/CC genotype, p = 0.016, see Table 4a. The prognostic effect of MTHFR A1298C might be caused by the neoadjuvant treatment as it could not be shown in primarily resected patients.

The survival of the neoadjuvantly treated and primarily resected patients are presented in detail in Table 4b and c. In neoadjvuantly treated patients the A1298C polymorphisms showed a survival benefit for the AA and CC genotypes, compared to the AC genotype (p = 0.02). In primarily resected patients the polymorphisms of both gene loci were not different in survival.

#### Survival in respect of tumor localization

**Figure 2:**
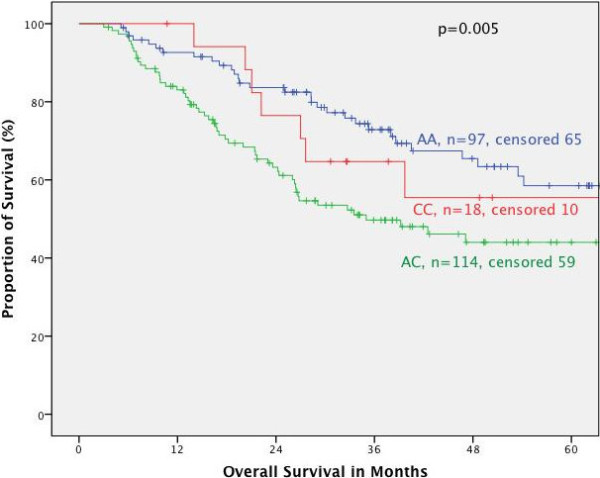
**Survival in gastric cancer patients according to UICC 6th edition, MTHFR A1298C.** Legend: Subgroup analysis for patients with adenocarcinoma of the esophagogastric junction type II, III and stomach dependent on polymorphisms of MTHFR A1298C. Median Survival times: AA 101.9 months, CC 74.2 months, AC 35.0 months, p = 0.005.

**Figure 3:**
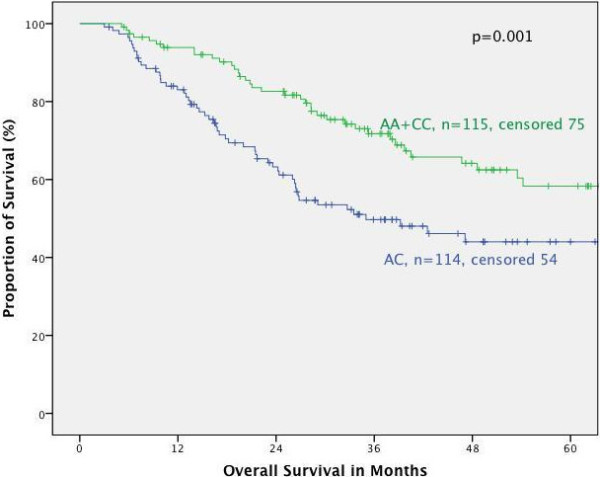
**Survival in gastric cancer patients according to UICC 6th edition, MTHFR A1298C AC versus AA/CC.** Legend: Subgroup analysis for patients with adenocarcinoma of the esophagogastric junction type II, III and stomach dependent on polymorphisms of MTHFR A1298C, comparing AC genoytpe to AA and CC genotype. Median Survival times: AA/CC 82.7 months, AC 35.0 months, p = 0.001).

**Figure 4:**
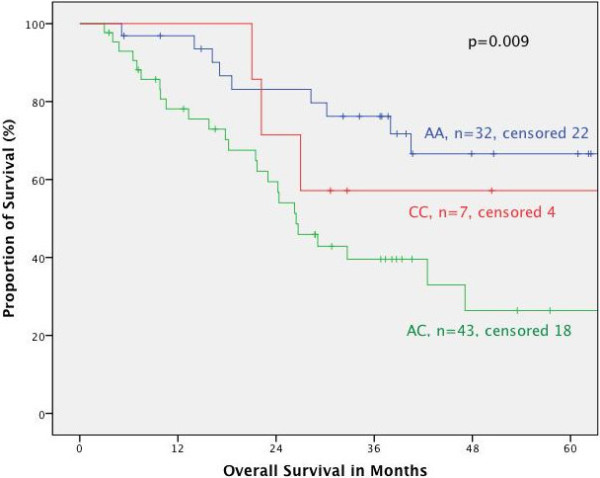
**Survival in gastric cancer patients according to UICC 7th edition, MTHFR A1298C.** Legend: Subgroup analysis for patients with adenocarcinoma of the stomach dependent on polymorphisms of MTHFR A1298C. Median Survival times: AA 82.7 months, CC not reached, AC 26.5 months, p = 0.009.

**Figure 5:**
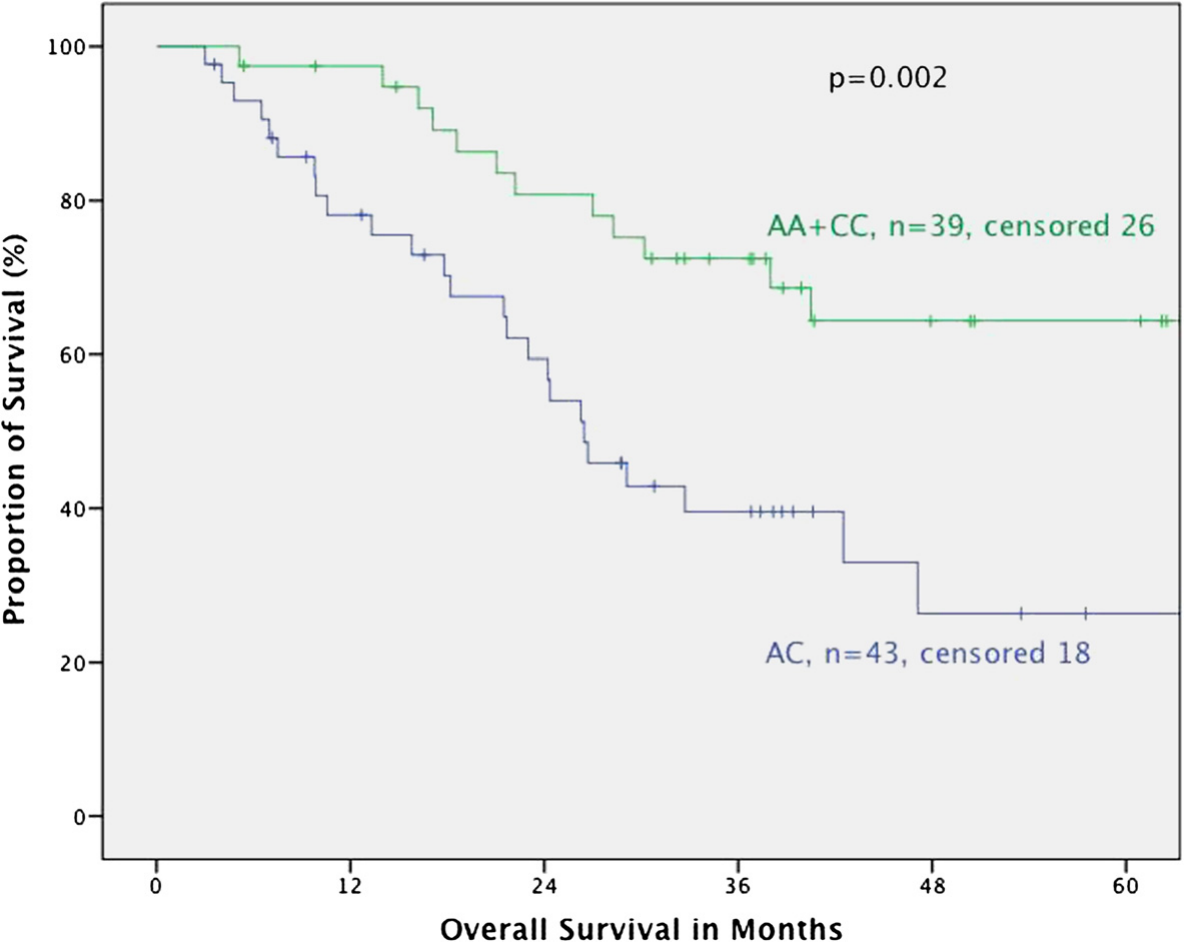
**Survival in gastric cancer patients according to UICC 7th edition, MTHFR A1298C AC versus AA/CC.** Legend: Subgroup analysis for patients with adenocarcinoma of the stomach dependent on polymorphisms of MTHFR A1298C, comparing AC genoytpe to AA and CC genotype. Median Survival times: AA/CC not reached, AC 26.5 months, p = 0.002.

Furthermore, the association of the respective genotypes with prognosis was tested stratified for the different tumor localizations. In primarily resected patients we found no correlation between MTHFR polymorphisms and prognosis for the different localizations. In neoadjuvantly treated patients both polymorphisms had no prognostic impact in adenocarcinomas of the esophagus (AEG I) nor if taken all junctional tumors (AEG I, II, III) together according to the UICC 7^th^ classification. This is in contrast to patients with adenocarcinoma of the stomach: according to the old classification (gastric cancer including AEG II, III, UICC 6^th^) (Figures 2 and 3) as well as in gastric cancer according to the new classification (gastric cancer without AEG II, III, UICC 7^th^) the MTHFR A1298C polymorphism was a prognostic factor (p = 0.005 and 0.009 respectively), (Figures 4 and 5). The AC genotype had a significantly worse prognosis compared to the rest in gastric cancer defined by the UICC6^th^ (p = 0.001, HR 2.0 (1.3-3.0)) and the UICC 7^th^ (p = 0.003, HR 2.8 (1.5-5.7)).

### Multivariate analysis

In neoadjuvantly treated gastric cancer patients according to UICC 6^th^ edition multivariate analysis (forward proportional hazard model) (including the univariate significant factors grading, pT-, pN-category, R-category, clinical and histopathological response, MTHFR A1298C polymorphisms as well as gender and age for adjustment) revealed pT-category (p = 0.002), R-category (p = 0.001), clinical response (p = 0.026) and MTHFR A1298C (p = 0.01) as independent prognostic factors. In gastric cancer patients according to UICC 7^th^ edition (including grading, pT-category, R-category, clinical response and MTHFR A1298C polymorphisms as well as gender and age) R-category (p = 0.001), clinical response (p = 0.021) and MTHFR A1298C (p = 0.028) were identified as independent prognostic factors (Tables 5 and 6). All prognostic factors were confirmed by the backward proportional hazard model.

**Table 5:** Multivariate analysis in neoadjuvantly treated patients with gastric cancer including AEG II, III (UICC 6th)

		**HR**	**95% CI**	**p**
**pT**	pT0	1.00		0.002
	pT1	2.16	0.2-20.8	
	pT2	5.10	0.7-37.5	
	pT3	9.80	1.3-74.3	
	pT4	14.50	1.7-122.9	
**R**	R0	1.00		0.001
	R1/2	2.20	1.3-3.5	
**Clinical response**	yes	1.00		0.026
	no	2.10	1.1-4.2	
**MTHFR A1298C**	AA	1.00		0.01
	AC	2.00	1.3-3.2	
	CC	1.49	0.7-3.3	

**Table 6:** Multivariate Analysis in neoadjuvantly treated patients with gastric cancer without AEG II, III (UICC 7th)

		**HR**	**95% CI**	**p**
**R**	R0	1.00		0.001
	R1/2	3.40	1.7-6.8	
**Clinical response**	yes	1.00		0.021
	no	10.30	1.4-75.5	
**MTHFR A1298C**	AA	1.00		0.028
	AC	2.80	1.3-5.9	
	CC	2.00	0.5-7.6	

## Discussion

Our study revealed the AC genotype of the MTHFR A1298C as a predictor of poor prognosis in patients with gastric cancer. However, this genotype was only a prognostic marker after neoadjuvant treatment not in primarily resected patients. This gives a clear hint towards the contribution of the chemotherapy on the prognostic impact of this polymorphism. Additionally the prognostic influence seems to be limited to gastric cancer (UICC 6^th^ or 7^th^ edition) since it was not apparent for adenocarcinomas of the esophagus.

Both examined MTHFR polymorphisms are known to be functionally relevant. The variants (CT and TT) of MTHFR C677T polymorphisms are associated with decreased activity of MTHFR, which results in higher homocystein levels and lower plasma folate levels [42,43]. Similarly, the A1298C variants (AC and CC) are associated with a lower enzyme activity, but results in literature are less final and conclusive than for C677T [31,32,44]. Our findings on MTHFR C677T are not statistically significant, but the trend for longer survival of the variants of C677T (CT and TT) would be in line with the functional hypothesis.

Furthermore MTHFR is thought to play an important role in response to fluoropyrimidine containing chemotherapy. A decreased activity of MTHFR results in higher 5,10-methylene tetrahydrofolate levels which leads to inhibition of thimidylate synthase and consequently to DNA damage [45-47] leading theoretically to an increased response and survival. This simplified theoretical approach cannot be confirmed by our data. Our results are in part conflicting, which points out the complexity of chemotherapy response and prognosis. Both seem to be affected by multiple pathways and related polymorphisms. In our study the AC variant of MTHFR A1298C is neither associated with response nor with improved outcome, but with poor prognosis in the subgroup of neoadjuvantly treated patients. However there exists data in literature, which correspond to our findings on the MTHFR A1298C polymorphism. Locally advanced adenocarcinoma of the stomach treated with cisplatin and 5-FU based chemotherapy with the variants of MTHFR A1298C (AC, CC) were associated with higher risks of recurrence and death in gastric cancer patients in a recent paper from our group [23] including 244 identical patients. Another study including unresectable, advanced gastric carcinomas reported similar results with shorter survival times of A1298C variants (AC and CC) (6.6 months median survival versus 18.5 months of the wild type (AA), p = 0.001) [48]. A pathway driven approach including amongst others both MTHFR polymorphisms showed conflicting results for esophageal cancer compared to ours. Longer survival and recurrence-free survival times could be shown for the MTHFR A1298C variants types (p = 0.01) as well as for combination of variant alleles at both loci (AC + CC) compared to the individuals with one wild type allele (AA) [49]. Two other studies reported a survival benefit for the TT variant of C677T [28,50] in patients with fluorouracil-based chemotherapy. In the adjuvant setting the CC genotype of MTHFR C677T was associated with shorter recurrence-free survival (p = 0.031) [28]. However, several other studies could not show any prognostic impact of one the MTHFR polymorphisms for patients with esophagogastric cancer [22,51-53], so that results remain controversial (see Table 7).

**Table 7:** MTHFR polymorphisms in literature

**Author**	**Year**	**Polymorphism**	**n**	**Treatment**	**Tumor entity**	**Response (p-value)**	**Prognosis (p-value)**	**Genotypes**	**In P F**
Ott [22]	2006	MTHFR C677T	235	neo CTx 135	AEGII/III/GC	0.14	0.14		
				OP 103	AEGII/III/GC	-	0.23		
Ott [23]	2011	MTHFR C677T	258	neo CTx	AEGI 114	n.s.	n.s.		
					AEGII-GC 144	n.s.	n.s.		
		MTHFR A1298C	258	neo CTx	AEGI 114	n.s.	n.s.		
					AEGII-GC 144	n.s.	0.02	AA > CC > AC	yes
Huang [28]	2008	MTHFR C677T	116	adj CTx	GC		0.04	TT/CT > CC	0.056
Wu [49]	2006	MTHFR C677T	210	neoCTx/RCTx	AEGI 174/SCC 36	n.a.	n.s.		
		MTHFR A1298C	210	neoCTx/RCTx	AEGI 174/SCC36	n.a.	0.011	AC/CC > AA	n.a.
Ruzzo [51]	2006	MTHFR C677T	175	pall CTx	GC	0.2	n.s.		
Chen [48]	2010	MTHFR A1298C	16/73	pall CTx	GC	-	<0.001	AA > AC/CC	n.a.
Lu [54]	2004	MTHFR C677T	75	pall CTx	GC	0.001	-	TT > CC/CT	<0.001
Goekkurt [52]	2009	MTHFR C677T	134	pall CTx	AEG/GC	0.214	0.319		
		MTHFR A1298C	134	pall CTx	AEG/GC	0.053	0.524		
Goekkurt [55]	2006	MTHFR C677T	52	pall CTx	GC	0.099	n.s.		
Shitara [50]	2010	MTHFR C677T	132	pall CTx	GC	n.a.	0.039	TT > CC/CT	
Lee [53]	2005	MTHFR C677T	40	adj CTx	GC		0.90		

neo = neoadjuvant, CTx = chemotherapy, RCTx = radiochemotherapy, OP = primarily resected, adj = adjuvant, pall = palliative, AEG = adenocarcinoma of the esophagus/esophagusgastric junction, GC = gastric cancer, n.s. = not significant, n.a. = not applied, In P F = independent prognostic factor.

As in our previous published studies [16,22,23] we could not show an association between the two polymorphisms and response to neoadjuvant treatment. Most of the studies on MTHFR polymorphisms and upper gastrointestinal cancer could not reproduce the association of response with fluoropyrimidine based chemotherapy having been described for other cancer types, mainly advanced colon carcinoma [45,56,57], apart from one Chinese study for the TT genotype of the MTHFR C677T polymorphism and response [54].

In one study including metastatic gastroesophageal adenocarcinomas, the AC variant of MTHFR A1298C was associated with lower response rates (27% responder in contrast to 48 and 46% responder for AA and CC genotype, p = 0.053) [52]. Other studies could not confirm the effect of MTHFR C677T and A1298C variants in response to chemotherapy [22,23,51] (see Table 7).

The conflicting and heterogenous findings highlight the demand on comprehensive pathway-based approaches for the prediction of response and prognosis by genetic polymorphisms as a potentially more successful and promising strategy. Beside the activity of the MTHFR, also the individual folate intake might influence outcome. Individuals with a high dietary folate intake are described to have a lower risk of developing gastrointestinal cancer. Consequently not only MTHFR polymorphisms, which play an important role in folate metabolism but also folate intake could be associated with prognosis in patients with esophagogastric carcinoma [50].

Further general problems of the presented studies are their heterogeneity and lack of comparability with respect to the number of patients included, inclusion criteria (type of tumor, tumor stage), different treatment concepts (palliative treatment versus curative treatment including radical surgery) and different genotypes. The change from UICC 6^th^ to 7^th^ might especially complicate the comparability of old and recent studies, as AEG II and III are classified differently [58]. To exclude this bias we repeated the analyses for both classifications and could show that in patients with AEG I or adenocarcinomas of the esophagogastric junction, the MTHFR polymorphisms did not have prognostic impact related to our recent paper [23].

Our study has some limitations. The first limitation is the inclusion of patients, on which we already reported recently, however, the follow-up was significantly extended [22,23]. Secondly it is a retrospective exploratory study and therefore has the typical disadvantages of this study type.

But in contrast to many other studies, we analyzed patients being primarily resected without perioperative concepts to evaluate the prognostic impact of the described polymorphisms apart from response to chemotherapy as a simple prognostic factor. In our study the prognostic impact of MTHFR A1298C could not be demonstrated in primarily resected patients. This leads to the conclusion that response to treatment plays an important role for influence on survival of the different genotypes, despite the effect on response could not be measured by a correlation with clinical and histopathological response in this study. A further strength of our study is the relatively high number of patients, to our knowledge the largest published series, the inclusion of adenocarcinomas only and a homogenous 5-FU containing preoperative treatment followed by resection.

## Conclusions

The AC genotype of the MTHFR A1298C was associated with a poor prognosis in neoadjuvantly treated gastric cancer patients, although there was no association with clinically or histopathologically assessed response to chemotherapy. This gives a clear hint towards the modulation of prognosis by chemotherapy, which cannot be measured by the available methods of response evaluation. Large patient numbers and pathway driven approaches seem necessary to evaluate the prognostic impact of polymorphisms in patients with esophagogastric adenocarcinomas to tailor treatment in the future.

## Abbreviations

MTHFR: Methylentetrahydrofolate reductase; AEG: Carcinoma of the esophagogastric junction; SNP: Single nucleotide polymorphism; GC: Gastric cancer; PCR: Polymerase chain reaction; TRG: Tumor regression grade.

## Competing interests

The authors declare that they have no competing interests.

## Authors’ contributions

SB participated in acquisition of data, statistical analysis, interpretation of data and manuscript writing and final approval of the manuscript; SK carried out the genotyping, participated in study design, interpretation of data and manuscript writing and final approval of the manuscript, GK participated in study design, interpretation of data and critical revision of the manuscript and final approval of the manuscript WW participated in interpretation of data, critical revision of the manuscript and final approval of the manuscript, FL participated in study design, interpretation of data, critical revision of the manuscript and final approval of the manuscript, RL participated in study design, interpretation of data, critical revision of the manuscript and final approval of the manuscript, CS participated in interpretation of data, critical revision of the manuscript and final approval of the manuscript, TB participated in statistical analysis, critical revision of the manuscript and final approval of the manuscript, KB participated in study design, interpretation of data, critical revision of the manuscript and final approval of the manuscript, RK carried out the genotyping, participated in study design, interpretation of data, critical revision of the manuscript and final approval of the manuscript, KO participated in study design, acquisition of data, interpretation of data, manuscript writing, critical revision of the manuscript and final approval of the manuscript. All authors have read and given final approval of the version to be published.

## Pre-publication history

The pre-publication history for this paper can be accessed here:

http://www.biomedcentral.com/1471-2407/14/58/prepub
